# Antiproliferative Effect of Bioaccessible Fractions of Four *Brassicaceae* Microgreens on Human Colon Cancer Cells Linked to Their Phytochemical Composition

**DOI:** 10.3390/antiox9050368

**Published:** 2020-04-28

**Authors:** Beatriz de la Fuente, Gabriel López-García, Vicent Máñez, Amparo Alegría, Reyes Barberá, Antonio Cilla

**Affiliations:** 1 Nutrition and Food Science Area, Faculty of Pharmacy, University of Valencia, Av. Vicent Andrés Estellés s/n, Burjassot, 46100 Valencia, Spain; 2 CIAM (Centro de Innovación Agronómica_Grupo Alimentario Citrus), Av. dels Gremis, Parcela 28. Pol. Ind. Sector 13, Riba-roja de Túria, 46394 Valencia, Spain

**Keywords:** microgreens, bioaccessible fractions, antiproliferative effect, colon cancer, Caco-2 cells, *Brassica*

## Abstract

The antiproliferative effect of the bioaccessible fractions (BFs) of four hydroponic *Brassicaceae* microgreens (broccoli, kale, mustard and radish) was evaluated on colon cancer Caco-2 cells vs. normal colon CCD18-Co cells after 24 h treatment with BFs diluted 1:10 *v/v* in cell culture medium. Their bioactivity was compared with the digestion blank, while the colon cancer chemotherapeutic drug 5-fluorouracil was used as a positive control. Cell viability (mitochondrial enzyme activity assay (MTT test) and Trypan blue test) and mechanisms related to antiproliferative activity (cell cycle, apoptosis/necrosis, mitochondrial membrane potential, reactive oxygen species (ROS) production, Ca^2+^ and glutathione (GSH) intracellular content) were studied. All microgreen BFs increased ROS and decreased GSH, altering the redox status and causing mitochondrial membrane dissipation followed by a general cell cycle arrest in G_2_/M and apoptotic cell death via a Ca^2+^-independent mechanism. As a result, the antioxidant bioactive compounds present in these microgreen species reduced the proliferation of tumoral cells (10 to 12.8% -MTT or 20 to 41.9% -Trypan blue), showing lesser effects with broccoli microgreens, in line with their lower ascorbic acid content and total antioxidant capacity. Therefore, the daily intake of microgreens within a balanced diet could be a preventive nutritional strategy to reduce the burden of chronic degenerative diseases such as colon cancer.

## 1. Introduction

*Brassica* vegetables represent one of the ten most economically important crops in the global agriculture and markets [1]. Cruciferous vegetables are good sources of fibre, vitamins, and minerals, whilst having a low lipid content; thus, these plant foods have traditionally been recommended in low-fat and heart-friendly diets [1,2]. In addition, different antioxidant bioactive compounds such as ascorbic acid, tocopherols, carotenoids, polyphenols and glucosinolates have been considered responsible for the prevention of chronic diseases attributed to cruciferous vegetables [3,4]. In this sense, the consumption of *Brassica* greens has been associated with a reduced risk of the development of colorectal, stomach, pancreatic, lung, breast and ovarian cancer [2,3,4,5]. A recent meta-analysis from observational studies concluded that the high intake of *Brassica* vegetables was negatively correlated with gastrointestinal cancer risk [6]. Moreover, The World Cancer Research Fund has pointed out that diets rich in cruciferous vegetables particularly protect against colon, rectum and thyroid cancers [7]. Regarding colon cancer, the third most common cancer worldwide, several epidemiological, experimental and clinical studies have considered vegetables belonging to the *Brassicaceae* family as one of the protective plant foods for this type of cancer [8]. 

In addition to the increasing scientific interest surrounding the relationship between diet and cancer, the population’s concerns about the effect of food in cancer prevention have led to the search for healthy products by both consumers and the food industry. Such is the case for the novel consumption of microgreens, which are considered functional food due to their important phytochemical content, which is usually even higher than their mature counterparts [5,9]. Recently, Choe et al. [5] have reported that microgreens present potential anti-cancer prevention because of their content of vitamins, carotenoids, polyphenols, and glucosinolates. However, as far as we are aware, there is no study in the literature about the bioactivity of *Brassica* microgreens. In this sense, only a few in vitro studies have shown antiproliferative effects on human colon cancer cell lines using extracts from broccoli sprouts [10], mature broccoli [11], kale in the adult stage [12], mature mustard leaves [13,14] and the edible parts (pod and flower) of radishes [15]. 

In this context, it is important to consider two relevant issues when studying the bioactivity of foods. Firstly, most of the in vitro cell culture studies with bioactive compounds use plant or food extracts. However, in recent years, the increasing importance of food matrices in the bioactivity of compounds has led to the use of food matrices together with simulated gastrointestinal digestion before studying cellular responses [16]. Secondly, in vitro pharmacological studies usually involve the direct exposure of cells to an isolated compound at high concentrations and/or long incubation times, avoiding the observation of possible synergistic effects (in a whole food matrix) between different compounds, as well as being uninformative for conclusions from a dietary point of view [17]. 

Taking the above into account, the aim of this study was to evaluate, for the first time, the antiproliferative effect of bioaccessible fractions (BFs) of broccoli, kale, mustard, and radish microgreens on human colon cancer Caco-2 cells, to obtain results closer to the in vivo situation with the use of a digested whole food matrix. 

## 2. Materials and Methods 

### 2.1. Chemicals

Dulbecco’s Modified Eagle Medium (D-MEM + GlutaMAX^™^ 4.5 g/L glucose); HEPES (1%, *v/v*); non-essential amino acids (1%, *v/v*); antibiotic solution (penicillin-streptomycin) (1%, *v/v*); antimycotic solution (fungizone) (0.2%, *v/v*); fetal bovine serum (FBS) (10%, *v/v*); trypsin-EDTA solution (2.5 g/L trypsin and 0.2 g/L EDTA); phosphate buffer solution (PBS) and distilled water were purchased from Gibco (Scotland, UK). Absolute ethanol and fuming hydrochloric acid 37% were supplied by Merck (Barcelona, Spain), while 2-propanol was from Scharlab (Barcelona, Spain). Dimethyl sulfoxide (DMSO); 2′,7′-dichlorofluorescein diacetate (DCFDA); 3-(4,5-dimethylthiazol-2-yl)-2,5-diphenyl-tetrazolium bromide (MTT); propidium iodide (PI); triton X-100; 5-fluorouracil (5-FU) and RNase A were obtained from Sigma Chemical Co. (St. Louis, MO, USA). An FITC-Annexin V Apoptosis Detection Kit I was provided from eBioscience (BD Biosciencies, CA, USA); 3,3′-dihexyloxacarboxycyanine iodide (DiOC_6_) was from Molecular Probes (Eugene, OR, USA); 5-chloromethylfluorescein acetate (Green CMFDA) was from Abcam (Cambridge, MA, USA) and fluo-3/acetoxymethyl (FLUO 3/AM) was from Santa Cruz Biotechnology (Heidelberg, Germany).

### 2.2. Cell Lines and Culture Conditions

The human colon cancer Caco-2 (HTB-37) and the normal human colon fibroblasts CCD18-Co (CRL-1459) cells were purchased from the American Type Culture Collection (Rockville, MD, USA). Both cell lines were cultured in 75 cm^2^ Falcon flasks (Thermo Scientific) in D-MEM and maintained in an incubator at 37 °C, 5% CO_2_ and 95% air atmosphere. 

For all cellular assays, the following common steps were carried out: (i) undifferentiated cells were harvested by trypsinization; (ii) cells were sown onto 24-well plates (Costar Corp., USA) at 10^5^ cells/cm^2^ and incubated with 1 mL of D-MEM for 24 h; and (iii) the medium was removed and cells were exposed to the different treatments (1 mL) for 24 h in an incubator. Then, cells were processed accordingly depending on the cellular assay. The incubation conditions were the same as for cell culture maintenance (37 °C/5% CO_2_/95% air atmosphere). All assays were performed with at least four technical replicates in two independent experiments. It should be noted that the BFs used in this study were obtained from four independent experiments that were further pooled and used in the independent cell culture assays.

### 2.3. Samples

BFs of four hydroponic *Brassicaceae* microgreens (broccoli, kale, mustard and radish) were obtained using the standardized static in vitro gastrointestinal digestion method derived from the Infogest Cost Action, as previously described by de la Fuente et al. [9]. In order to avoid the presence of residues from the simulated gastrointestinal digestion that could affect the results of cellular tests, BFs were previously treated. They were homogenized and centrifuged (5 min at 4000 rpm), and the supernatants were filtered (0.22 µm). Filtered samples were diluted with D-MEM (1:1, 1:2, 1:5, 1:10 and 1:20 *v/v*) and their pH and osmolality were measured. Dilutions within the physiological range of pH (7–7.5) and osmolality (280–330 mOsm/L) were selected (1:5, 1:10 and 1:20 *v/v*) for the preliminary tests of cell viability on Caco-2 and CCD18-Co cells. According to cell viability tests, the 1:10 dilution was considered optimal and was used for cellular assays. In order to avoid the overestimation of the results, the values of the BFs of microgreens were compared with those of the digestion blank to remove any activity caused by digestive enzymes or simulated fluids on cells. In addition, the chemotherapy drug used against colon cancer, 5-FU, at 25 µM [18] was applied as a positive control and compared to the D-MEM culture medium as a negative control (untreated cells). 

Table 1 shows the main antioxidant bioactive compounds (ascorbic acid, total soluble polyphenols, total carotenoids and total isothiocyanates) as well as the total antioxidant capacity (TEAC and ORAC methods) present in the BFs from the four microgreens diluted 1:10 (*v/v*), calculated from previous data [9].

### 2.4. Cell Viability Assays

Mitochondrial enzyme activity assay (MTT test) and the Trypan blue test were used to evaluate cell viability as previously described [19]. The first method was applied on both tumoral (Caco-2) and non-tumoral (CCD18-Co) colon cells to verify that the effect of the BFs of microgreens only affected tumoral cells, while the second method was used as a complementary assay to confirm the estimated antiproliferative effect on Caco-2 cells. 

#### 2.4.1. Mitochondrial Enzyme Activity 

The MTT assay is based on the ability of mitochondrial dehydrogenase enzymes to reduce the tetrazolium ring of MTT, resulting in purple formazan crystals that are colorimetrically measurable. Briefly, the culture medium or tested samples were removed from the wells and 1 mL of MTT (0.5 mg/mL in PBS) was added to the cells before they were incubated for 2 h. Then, the MTT solution was discarded and formazan was dissolved with 1.5 mL of a solution of acidic 2-propanol (0.1N HCl, 0.1% *w/v* triton). The formazan product was measured by spectrophotometry (Perkin Elmer, lambda 2 UV-VIS) at 570 nm, with background subtraction at 690 nm. The results were expressed as a percentage of cell viability with respect to the digestion blank.

#### 2.4.2. Trypan Blue Exclusion Test

The Trypan blue exclusion test allows us to differentiate visually between living (clear) and dead (blue) cells since the dye only can permeate through the damaged membrane of non-viable cells. The culture medium or tested samples were removed from the wells and Caco-2 cells were harvested by trypsinization and diluted with D-MEM up to 1 mL. Then, cell suspensions were mixed with Trypan blue dye (1:1) and approximately 20 µL was added to a cell counting chamber to check cell viability (Countess^®^ II Automated Cell Counter, Thermo Fisher Scientific). The results were expressed as a percentage of cell viability with respect to the digestion blank.

### 2.5. Measurement of Mechanisms Involved in Cell Death

In order to elucidate the mechanisms that cause the cellular antiproliferative effect of the BFs of microgreens on Caco-2 cells, different tests were carried out by using flow cytometry (FACSVerse, BD Biosciencies). For all assays, at least 10,000 events were analyzed for each sample. 

#### 2.5.1. Cell Cycle Analysis

The cell cycle distribution was monitored by quantifying the cellular DNA through staining with PI (λexc = 351 nm and λem = 617 nm) according to Cilla et al. [20]. Briefly, floating cells were taken from the culture medium and collected in cytometer tubes, while adherent cells were washed with PBS, trypsinized and transferred into cytometer tubes. Cell suspensions (floating + harvested cells) were centrifuged (5 min at 1500 rpm) and suspended in 100 µL PBS. Then, cells were fixed in cold ethanol–PBS (70:30, *v/v*) and treated with 50 µL of RNAase A (100 µg/mL) and 50 µL of PI solution (40 µg/mL) in an incubator for 30 minutes. The results were expressed as the percentage of cells in each cell phase stage.

#### 2.5.2. Detection of Cellular Apoptosis

The different apoptotic stages were determined using the FITC-Annexin V Apoptosis Detection Kit I (BD Biosciences, CA, USA) in conjunction with the PI fluorochrome, according to the instructions of the manufacturer. Briefly, the culture medium or tested samples were removed from the wells, cells were washed with 1 mL of cold PBS before being harvested by trypsinization and transferred to cytometer tubes where they were centrifuged at 900 rpm for 5 min. Supernatants were discarded and cell pellets were re-suspended in 100 µL of binding buffer (diluted 1/10 with distilled water, *v/v*). Then, 5 µL of annexin V and 5 µL of PI solution were added and the mixture was incubated at room temperature in darkness for 15 minutes. After incubation, 400 µL of binding buffer was added and samples were analyzed by flow cytometry (λ_exc_ = 488 nm and λ_em_ = 525 for annexin V and λ_exc_ = 351 nm and λ_em_ = 617 for PI) using the appropriate two-dimensional gating method. The results were expressed as the percentage of cells in each cell death stage.

#### 2.5.3. Intracellular Reactive Oxygen Species (ROS) Production

The intracellular accumulation of reactive oxygen species (ROS) was determined according to Cilla et al. [20], with some modifications. Both floating and harvested cells were transferred to a cytometer tube and centrifuged 5 min at 1500 rpm. Cell pellets were suspended in 500 µL of D-MEM and 500 µL of PBS. Then, cell suspensions were treated with 20 µL of 500 µM DCFDA and incubated for 30 min in darkness. After the incubation period, samples were centrifuged again (5 min at 900 rpm) and cells were suspended in 300 µL of PBS. The oxidation of DCFDA to fluorescent dichlorofluorescein (λexc = 495 nm and λem = 529 nm) was determined by flow cytometry. The results were expressed as the fluorescence intensity in arbitrary units.

#### 2.5.4. Intracellular Glutathione (GSH) Determination

The intracellular glutathione GSH content was analyzed by using the Green CMFDA fluorochrome [21] with some modifications. Both floating and harvested cells were transferred to a cytometer tube and centrifuged for 5 min at 1500 rpm. Cells were suspended in 445 µL of PBS and 5 µL of green CMFDA (100 µM) was added. Immediately after, the tubes were incubated for 40 min in darkness. Cellular suspensions were centrifuged (5 min/800 rpm/24 °C) and the cell pellets were dissolved in 500 µL of PBS. The emitted fluorescence was measured by flow cytometry (λ_exc_ = 492 nm and λ_em_ = 516) and is proportionally related to the reduced intracellular GSH. The results were expressed as a percentage of intracellular glutathione.

#### 2.5.5. Mitochondrial Membrane Potential Changes (ΔΨM)

The lipophilic cationic fluorescent dye DiOC6 was used to measure the mitochondrial transmembrane potential [20] with some modifications. Briefly, both floating and trypsinized cells were transferred to cytometer tubes. Next, 4 µL of 10 µM DiOC_6_ were added and the mixture was incubated for 15 min in darkness. After centrifugation (5 min at 900 rpm), supernatants were removed, and cells were suspended in 300 µL of PBS. The fluorescence was measured at λexc = 485 nm and λem = 499 nm by flow cytometry, where loss of cellular fluorescence is related to loss of cell membrane integrity. The results were expressed as a percentage of mitochondrial membrane depolarization with respect to the digestion blank.

#### 2.5.6. Intracellular Calcium (Ca^2+^) Content

The intracellular Ca^2+^ level was evaluated by using the Fluo-3/AM fluorochrome [20], with some modifications. Briefly, both floating and harvested cells were transferred to a cytometer tube and centrifuged 5 min at 1500 rpm. Cell pellets were suspended in 500 µL of PBS and 10 µL of Fluo-3/AM solution (100 µM) was added in darkness conditions. Cells were incubated for 40 minutes, centrifuged at 800 rpm for 5 min and suspended in 300 µL of PBS. The fluorescence emitted after Fluo-3/Ca^2+^ blinding was measured by flow cytometry (λ_exc_ = 506 nm and λ_em_ = 526). The results were expressed as fluorescence intensity in arbitrary units.

### 2.6. Statistical Analysis

A one-way analysis of variance (ANOVA) followed by the Tukey’s multiple range test were applied to determine statistically significant differences (*p* < 0.05) among the different samples. All analyses were performed with the software Statgraphics Plus 5.1 (Statpoint Technologies Inc., Warrenton, VA, USA).

## 3. Results and Discussion

Among the variety of bioactive constituents present in cruciferous vegetables, polyphenolic compounds and especially glucosinolates have been considered as the major constituents responsible for their biological activities [3]. Glucosinolates are sulfur-containing compounds almost exclusively found in *Brassicaceae* family plants [1]. They are precursors to isothiocyanates, compounds with anti-tumor activity, and affect multiple pathways [22]. A previous study by our research group showed that both total soluble polyphenols and total isothiocyanates were the main compounds responsible for the total antioxidant capacity after digestion for broccoli, kale, mustard, and radish microgreens (Table 1). Their BFs could act at a cellular level through one or more of the mechanisms described for these phytochemicals on human colon cancer cells (induction of phase II enzymes, apoptosis, cell cycle arrest, oxidative stress, MAPK signaling, autophagy, and changes in gut microbiota) [22,23].

### 3.1. Antiproliferative Effect of Microgreen’s Bioaccessible Fractions on Tumoral Cells

The results of cell viability after treating non-tumoral and tumoral human colon cells for 24 h with microgreen BFs are reported in Table 2. In non-tumoral cells (CCD18-Co), samples exerted no antiproliferative effect, except for mustard BF, while in Caco-2 cells microgreen BFs produced a moderated cell viability reduction that ranged between 10–12% with respect to the blank digestion, as determined by the MTT test. In addition, the BFs of broccoli, kale and radish showed a significant (*p* ˂ 0.05) antiproliferative action in tumoral vs. non-tumoral cells. The antiproliferative effect observed on tumoral cells was confirmed through the Trypan blue exclusion test, obtaining higher cell viability reductions for all samples (20–42%) and being stronger in kale ≥ radish = mustard = broccoli (data not shown). As expected, the drug 5-FU showed a higher cell growth inhibition on tumoral cells (23%) vs. non-tumoral cells (14%) (see Table 2 footnotes).

As far as we know, there are no previous studies in the literature that evaluate the antiproliferative effect of microgreens subjected to a gastrointestinal in vitro digestion in colon cancer cells. However, the activity of extracts from broccoli, kale, mustard and radish in different growth stages on human colon cancer cells has been studied by some authors. Le et al. [10] demonstrated that 5-day-old broccoli sprout extract (0.063–0.500 mg/mL) produced cell growth inhibition in Caco-2 cells in a dose-dependent manner, with an IC_50_ of 0.189 mg/mL at 48 h. Ferrarini et al. [11] observed that extracts from fresh mature broccoli at concentrations between 0.1–1 g fresh weight/mL reduced cell proliferation by 28–68% in HT-29 cells treated for 24 h. In addition, mustard leaf extracts (*Brassica juncea)* at concentrations of 50–100 µg/well for 48 h, inhibited HT-29 cell growth by 32–36% (MTT test) [13], which correlates with the results observed in our study using a Trypan blue test (29%). The authors suggested that the content of bioactive compounds such as chlorophyll, carotenoids, glucosinolates and vitamin C could have induced the cell growth inhibition. Likewise, curly kale (*Brassica oleracea* L. convar. *Acephala* var. *Sabellica*) extracts from green and red varieties inhibited Caco-2 cell proliferation (MTT test) by 30–70% when they used extracts containing a concentration of total polyphenols between 50–150 mg GAE/L fresh weight [12]. In our case, by transforming our results of total polyphenols in kale BF from dry weight to fresh weight and considering the 1:10 ***v/v*** dilution used for the cells, an approximate value of 68 mg GAE/L fresh weight is obtained, inducing a decrease in viability of 42% (Trypan Blue) within the range shown by the previous authors. As for radish, high cytotoxicity against HCT116 cells (IC_50_ of 9.42 ± 0.46 μg/mL) was observed in pod and flower extracts from young Thai rat-tailed radish [15].

Using different cell lines than those of colon cancer, only one study, using BFs from broccoli sprouts (*Brassica oleracea* L. cv. *Italica*), reported that treatment with 0.1–1 mg/mL for 72 h is effective against cell proliferation in prostate cancer cells (AT-2 y MAT-Lylu), leading to a cell growth inhibition of 26–39% [24], in line with the results in the present study. Although no data have been found in the literature on radish leaf bioactivity in intestinal cells, Li et al. [25] reported the antiproliferative effects of aqueous extracts from three-day-old radish sprouts for 72 h at 0.1 mg extract/mL (with 20.4 µM of sulforaphane) on human non-small lung cancer H1299 and HCC817 cells. These results are higher than the use of sulforaphane alone at the same concentration, possibly due to the presence of other isothiocyanates in the extracts.

### 3.2. Cell Cycle Analysis

**Figure 1:**
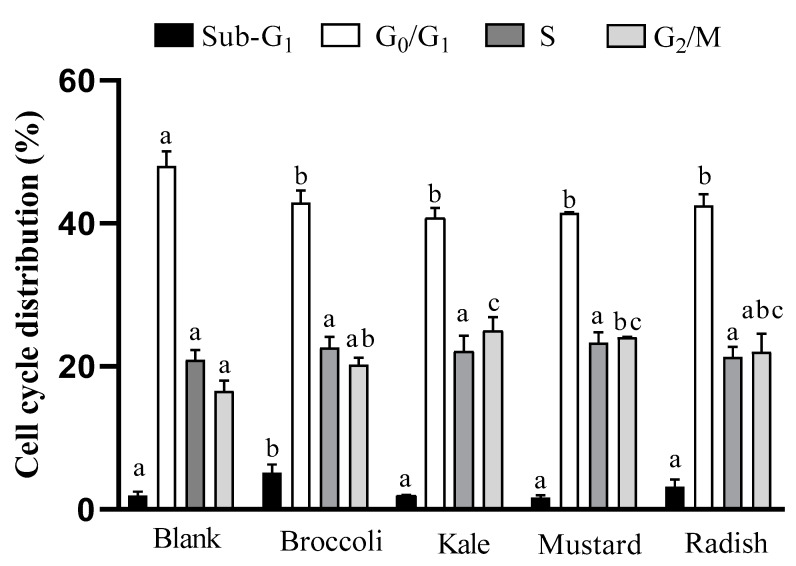
Evaluation of cell cycle distribution after incubation for 24h with bioaccessible fractions obtained from *Brassicaceae* microgreens (diluted 1:10 ***v/v*** in DMEM) in Caco-2 cells. Results are expressed as mean ± standard deviation (n = 4) in two independent assays. Different lowercase letters (a–c) indicate statistically significant differences (*p* < 0.05) among samples within the same cellular phase. Positive control (5-FU) increased 8-fold proportion of cells in sub-G_1,_ with a concomitant decrease in the rest of phases (G_0_/G_1_, S and G_2_/M) vs. control (untreated cells).

The cell cycle is a sequence of organized and monitored events within the process of cell division and proliferation. Cancer cells are characterized by uncontrolled cell proliferation, usually linked to an alteration of cell cycle checkpoints. In this sense, several natural compounds and chemotherapeutics can inhibit the cell growth of cancer cells, inducing arrest in different cell cycle phases (G_0_/G_1_, S and G_2_/M) [26,27]. The effect of microgreen BFs on Caco-2 cell cycle distribution after 24 h of treatment is shown in Figure 1. 

All samples induced a significant decrease (*p* ˂ 0.05) in the G_0_/G_1_ phase (cellular growth) regarding the digestion blank, which is associated with a lower rate of cell proliferation. A significant arrest (*p* ˂ 0.05) in G_2_/M phase with respect to the digestion blank for kale and mustard was also observed. Besides, there was a statistically significant increase in the sub-G_1_ phase (related to apoptosis) for broccoli. On the other hand, the positive control 5-FU increased the proportion of cells in the apoptotic phase (sub-G_1_) by eight times and reduced the proportion of cells in the rest of the cell cycle phases (G_0_/G_1_, S y G_2_/M) in comparison with the untreated cells. 

Similar results were observed with a five-day-old broccoli sprout extract (0.2 mg/mL for 48 h) since it provoked an increase in sub-G1 phase and an arrest in G2/M phase (~7 times and ~3 times vs. control, respectively) in Caco-2 cells [10]. The results are also consistent with the antiproliferative effect described for sulforaphane (15 µM for 24h), an isothiocyanate present in all BFs of microgreens, which produced an increase in sub-G1 and G_2_/M phases in HT-29 colon cancer cells [28]. Moreover, these results are also in agreement with the increase in sub-G_1_ and G_2_/M arrest phases in prostate cancer cells (PC-3), as induced by mustard (*Brassica juncea*) sprout extracts (10–100 μg/mL) after 12 h treatment [29]. Accordingly, these studies suggest that an increase in the G_2_/M phase, induced by the specific combinations of antioxidant bioactive compounds present in the microgreen BFs, could be a preceding event to apoptosis in tumor cells.

### 3.3. Apoptotic Pathway Activation

**Figure 2:**
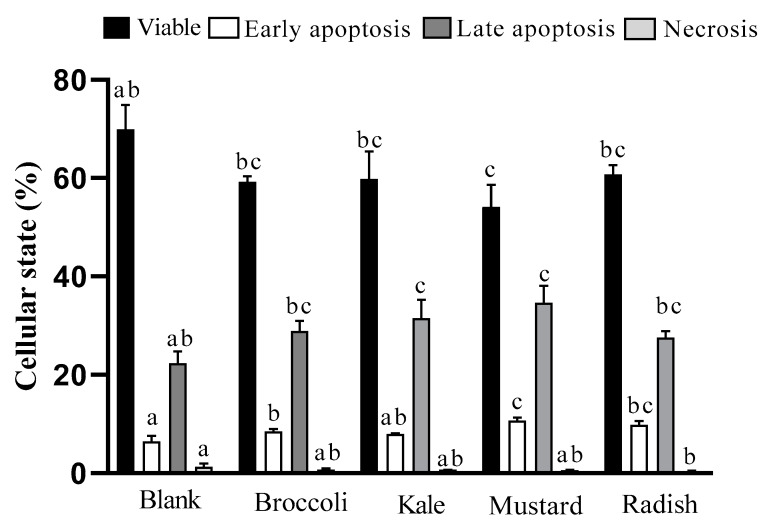
Effect of the incubation of bioaccessible fractions obtained from *Brassicaceae* microgreens (diluted 1:10 ***v/v*** in DMEM) for 24 h on cell death in Caco-2 cells. Results are expressed as mean ± standard deviation (n = 4) in two independent assays. Different lowercase letters (a–c) indicate statistically significant differences (*p* < 0.05) among samples within the same cellular state. Positive control (5-FU) decreased proportion of viable cells (74.8 to 40.6%) and increased apoptotic cells in late stage (17 to 53%) vs. control (untreated cells).

Some relevant pro-apoptotic events such as the depolarization of mitochondrial transmembrane potential, the externalization of plasma membrane phosphatidylserine, and increase in intracellular calcium levels were determined in order to evaluate apoptosis activation after treatment with microgreen BFs. Annexin V-FITC staining is a common procedure used to detect phosphatidylserine externalization, an event in the early phases of apoptosis, which triggers protease activation (Annexin V+). Its combination with PI (DNA binding compound), allows us to distinguish between the apoptotic viable and dead cells (late apoptosis), which show nuclear cell membrane deterioration (Annexin V+/PI+). However, the nuclear cell membrane could be damaged independently of apoptosis by processes such as necrosis (Annexin V−/PI+) [30]. The results of the induction of apoptosis and/or necrosis by the microgreen BFs are shown in Figure 2. 

The BFs of broccoli, mustard and radish microgreens showed an increase in early apoptosis (25–66%) while BFs of kale and mustard presented a rise in late apoptosis (25–55%) vs. the digestion blank. In addition, for all microgreen digests, no cell death was detected by necrosis with respect to the digestion blank, ranking the samples mustard ≥ kale = radish = broccoli. This observation agrees with the results of cell viability and arrest in the G_2_/M phase of the cell cycle previously shown. Likewise, the drug 5-FU showed a significant (*p* ˂ 0.05) and important decrease in viable cells (75% to 41%) as well as an increase in late apoptotic cells (17% to 53%) with respect to the untreated cells. 

In line with our study, it has been described that sulforaphane shows an antiproliferative effect in HT-29 colon cancer cells at 15 μM during 24h of treatment with a 75% decrease in cell viability and induction of apoptosis cell death excluding the necrotic pathway [28]. Moreover, raw mustard (*Brassica juncea*) leaf extracts induced chromatin condensation, nuclear fragmentation and apoptotic body formation in HT-29 cells after 24 hours of treatment (100 μg/well) [13]. Similarly, extracts of a fresh green kale variety (*Brassica oleracea* L. convar. *Acephala* var. *Sabellica*) with treatments containing concentrations of total polyphenols between 25–75 mg GAE/L fresh weight for 4h increase apoptosis in Caco-2 and HT-29 cells between 40–70% compared to the control [12]. In our study, the BF of kale with 1:10 dilution (~68 mg GAE/L fresh weight) shows a 33% increase in apoptosis (sum of early and late apoptosis stages and necrosis) compared to the digestion blank, given that a slightly lower percentage was described by the previous authors.

**Figure 3:**
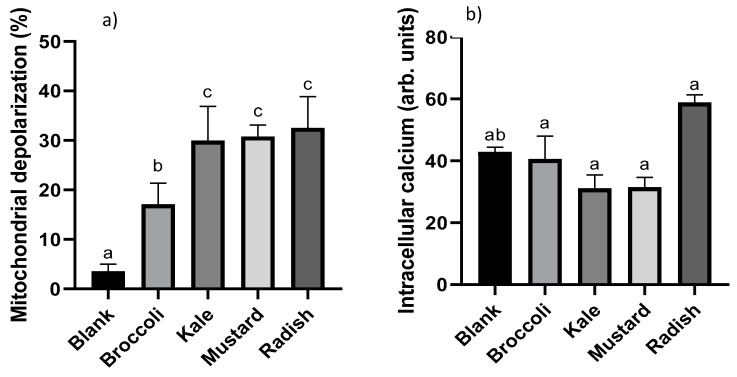
Evaluation of the changes in the mitochondrial membrane potential (**a**) and intracellular calcium levels (**b**) produced after incubation for 24 h with bioaccessible fractions obtained from *Brassicaceae* microgreens (diluted 1:10 ***v/v*** in DMEM) in Caco-2 cells. Results are expressed as mean ± standard deviation (n = 4) in two independent assays. Different lowercase letters (a–c) indicate statistically significant differences (*p* < 0.05) among samples. Positive control (5-FU) produced a marked mitochondrial depolarization (6-fold) and raise of intracellular Ca^2+^ levels (64%) compared to control (untreated cells).

Mitochondria is one of the most important organelles involved in the apoptotic pathway activation, and its internal membrane depolarization is considered a point of no return, which leads to ROS overproduction and ultimately cell death [31]. Mitochondrial membrane depolarization can be estimated by using the cation DiOC_6_, which is preferentially accumulated and retained in mitochondria of intact cells due to their large negative membrane potential. Mitochondrial membrane potential disruption leads to the release of this compound out of the cells with the subsequent loss of fluorescence, which can be measured by flow cytometry [32]. The results of the percentage of depolarized mitochondrial cells after 24 h of treatment with the microgreen BFs are shown in Figure 3a. The percentage of depolarization ranged from 17% to 32%, showing a statistically significant increase (*p* ˂ 0.05) in depolarized mitochondrial cells for all treatments. Broccoli had less capacity to modify the membrane potential of the mitochondria compared to kale, mustard and radish samples. Maybe the lower total antioxidant capacity and vitamin C content in the broccoli sample could explain the differences observed (see Table 1). The positive control (5-FU) showed a clear six-fold induction in the dissipation of the membrane potential with respect to the untreated cells. Le et al. [10] observed that the treatment with five-day-old broccoli (*Brassica oleracea* L. var. *italica*) sprout extracts at 0.2 mg/mL for 3–48 h notably increased depolarized mitochondrial cells by 42% to 80% in a time-dependent manner. Moreover, authors observed that the broccoli extract increased cell population in sub-G_1,_ confirming the activation of the apoptotic pathway. Similarly, Chaudhary et al. [33] described the antiproliferative effect of broccoli (*Brassica oleracea italica* L. var. Plenck) sprout extracts at 10–100 μg/mL for 12 h in prostate cancer cells (PC-3) with a slightly higher depolarization of the mitochondrial membrane potential (37–42%) than in our present study.

Another important apoptotic trigger is the increase in intracellular calcium in the cells, which is involved in the mitochondrial membrane potential disruption and release of pro-apoptotic molecules (cytochrome C and endonuclease G) [34]. Calcium level oscillations in live cells can be measured by permeable chemical indicators such as Fluo-3/AM. This compound sharply increases its fluorescence in the presence of calcium and can be measured by flow cytometry [35]. The intracellular Ca^2+^ in Caco-2 cells after 24 h of treatment with the microgreen BFs is shown in Figure 3b. The values ranged from 31% to 59% but there were no statistically significant differences (*p* > 0.05) with respect to the digestion blank; thus, cell death occurs through a non-calcium-dependent process. The BF of radish showed a non-significant (*p* > 0.05) increase tendency in the calcium concentration, which could be due to the higher total antioxidant capacity (TEAC method) and the higher total isothiocyanate content in the BF of radish microgreen (Table 1). On the other hand, the positive control (5-FU) produced a marked increase in calcium intracellular concentration (64%) compared to untreated cells.

### 3.4. Cellular Redox Disruption

**Figure 4:**
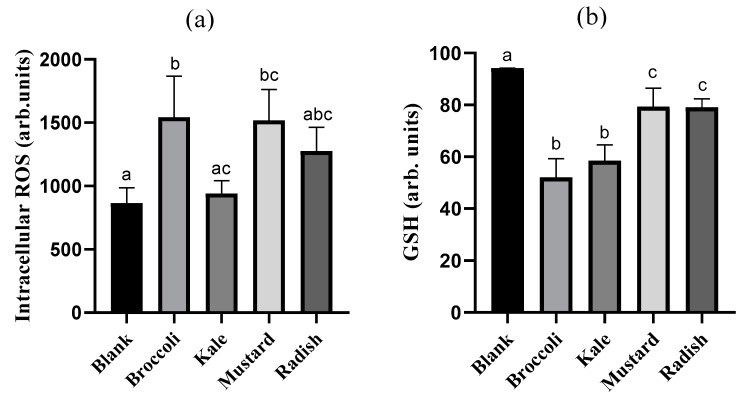
Impact of bioaccessible fractions obtained from *Brassicaceae* microgreens (diluted 1:10 *v/v* in DMEM) on (**a**) reactive oxygen species (ROS) and (**b**) glutathione (GSH) in Caco-2 cells after incubation for 24h. Results are expressed as mean ± standard deviation (n = 4) in two independent assays. Different lowercase letters (a–c) indicate statistically significant differences (*p* < 0.05) among samples. Positive control (5-FU) increased intracellular ROS (84%) and reduced GSH levels (24%) compared to control (untreated cells).

In order to investigate the implication of cellular redox alteration on the antiproliferative effect produced by microgreen BFs, intracellular ROS and GSH levels were evaluated. The intracellular ROS content in Caco-2 cells after 24 h treatment with the BFs of microgreens is reported in Figure 4a. Only broccoli and mustard BFs showed a statistically significant increase (*p* ˂ 0.05) in ROS content (94% and 62%, respectively) with respect to the digestion blank. The fact that broccoli and mustard have the lowest content of specific antioxidant bioactive compounds as vitamin C and total antioxidant capacity, and total soluble polyphenols, respectively (see Table 1), could explain the higher ROS intracellular content observed with these samples in tumoral cells. The positive control (5-FU) also showed a clear intracellular accumulation of ROS (84%) vs. untreated cells. Similar results have been observed in 3 and 5 days-old mustard (*Brassica juncea*) and three-, five- and seven-day-old broccoli (*Brassica oleracea italica*) extracts in the PC-3 prostate tumoral cells. Treatment for 12 h with broccoli (48–80 µg/mL) and mustard (44–116 µg/mL) extracts produced a strong ROS overproduction of 2.6- to 5.5-fold and 2.7- to 3.7-fold vs. untreated cells with a concomitant increase in apoptotic cells, suggesting that the ROS unbalance could be an important trigger of the apoptotic pathway [29,33]. 

The intracellular GSH content in Caco-2 cells after 24 h of treatment with the microgreen BFs is shown in Figure 4b. All BFs of microgreens reduced the GSH content in Caco-2 cells relative to the digestion blank in a statistically significant way (*p* ˂ 0.05) (15–42%), being higher in broccoli and kale. On the other hand, the 5-FU (positive control) also showed a significant decrease in intracellular GSH (24%) respect to the untreated cells. Recent investigations have demonstrated that some bioactive compounds such as polyphenols can block the antioxidant defense of tumor cells decreasing the intracellular GSH content and inhibiting the expression of antioxidant enzymes. However, the same compounds often exert both antioxidant and pro-oxidant properties, depending on concentration used, cell type, exposure time and environmental conditions [36]. The huge variability in the content of bioactive compounds as well as their composition and total antioxidant capacity among microgreens could explain the differences in the GSH content observed. We hypothesized that the lower vitamin C content and total antioxidant capacity for broccoli as well as the lower carotenoid and isothiocyanate total content in kale could be responsible for generating a greater oxidative environment and subsequent consumption of GSH. There is no data in the literature in this regard about microgreens.

Since microgreens of broccoli, kale, mustard and radish have shown an antiproliferative effect on colon cancer cells after being subjected to a simulated gastrointestinal digestion, this study provides pre-clinical scientific evidence to recommend their consumption as part of a balanced diet for the prevention of colon cancer. In addition to a nutritional and public health point of view, the knowledge of the mechanisms involved in cell death could serve as a starting point for further studies on the effect of the combination of these microgreens or their bioactive compounds as co-adjuvants in the treatment of colon cancer patients. In this sense, Varghese et al. [37] suggested that conventional therapy combined with whole plant foods or different molecules from plants with proven cytotoxic activities, could affect more cancer cell signaling pathways than a single compound in cancer treatment. On the other hand, malignant tumors such as colon cancer often create new tumor cell lines, thus resulting in highly variable sensitivity to therapy and the resistance of some cell lines to treatment [38,39]. The simultaneous administration of common synthetic drugs and plant-derived molecules should enhance the effectiveness of the chemotherapeutic substances, as well as postpone the development of drug resistance [37]. In this context, Redondo-Blanco et al. [40] reviewed several studies based on combinatory effects of chemotherapy drugs and bioactive compounds from plants. They concluded that these combinations might achieve improvements in the total or partial remission of colorectal tumors and minimize the possible side effects of drug treatment or radiotherapy due to the fact that plant molecules could reinforce the drug effective dose or modulate diverse target pathways in cancer cells. Given that this study shows that BFs of microgreens can induce apoptosis in Caco-2 cells, further investigations could be focused on determining the apoptotic pathways activated through the evaluation of the protein level and phosphorylation status of the Bcl-2 family members and activation of caspases. Furthermore, the identification of the specific molecules responsible for this anticancer activity would be required for their possible use in a combinational clinical approach in oncology.

## 4. Conclusions

The bioaccessible fractions of broccoli, kale, mustard and radish *Brassicaceae* microgreens showed a statistically significant (*p* ˂ 0.05) antiproliferative effect in the colon cancer Caco-2 cells, whilst a lower effect was observed in the case of broccoli microgreens, in line with their lower ascorbic acid content and total antioxidant capacity. This in turn indicates that differences in the bioaccessibility of antioxidant bioactive compounds could be a useful tool to select samples with higher bioactivity linked to their phytochemical content in pre-clinical models for subsequent assays in humans. The bioactivity of edible microgreens after the in vitro gastrointestinal digestion process occurred through a concatenation of different events: (i) the alteration of the redox cellular state by ROS increase and GSH decrease (non-dependent on intracellular calcium accumulation); (ii) the arrest of the cell cycle in the G_2_/M phase and a reduction in the proportion of cells in the G_0_/G_1_ phase; (iii) the dissipation of mitochondrial membrane potential; and (iv) the induction of programmed cell death via the mitochondrial pathway (absence of necrosis). It is worth noting that the consumption of fresh *Brassica* microgreens within a balanced diet could be an effective nutritional strategy in colon cancer prevention and possibly other gastrointestinal and non-gastrointestinal cancers.

## Figures and Tables

**Table 1:** Concentration of antioxidant bioactive compounds and total antioxidant capacity present in the bioaccessible fractions of microgreens used for antiproliferative effect in Caco-2 cells (diluted 1:10 ***v/v*** in Dulbecco’s Modified Eagle Medium (D-MEM)), calculated from previous data [9].

	Bioaccessible Fraction			
Parameter	Broccoli	Kale	Mustard	Radish
**Bioactive compound**				
Ascorbic acid (mg/100 g FW)	0.056 ± 0.04 ^b^	0.105 ± 0.04 ^a^	0.114 ± 0.05 ^a^	0.119 ± 0.04 ^a^
Total carotenoids (mg β-carotene/100 g DW)	0.018 ± 0.01 ^b^	0.012 ± 0.01 ^c^	0.025 ± 0.01 ^a^	0.023 ± 0.02 ^a^
Total isothiocyanates (mg sulforaphane/100 g DW)	20.45 ± 4.79 ^b^	20.72 ± 1.03 ^b^	24.89 ± 2.57 ^b^	51.29 ± 3.39 ^a^
Total soluble polyphenols (mg GAE/100 g DW)	142.79 ± 17.5 ^a^	144.77 ± 14.01 ^a^	82.06 ± 3.10 ^b^	143.48 ± 6.23 ^a^
**Total antioxidant capacity**				
ORAC (µM Trolox Eq/100 g)	364.5 ± 28.12 ^b^	739.15 ± 11.62 ^a^	745.25 ± 70.16 ^a^	525.89 ± 72.17 ^b^
TEAC (µM Trolox Eq/100 g)	7.84 ± 0.91 ^c^	9.87 ± 1.13 ^b^	11.08 ± 1.86 ^b^	13.77 ± 1.13 ^a^

Fresh weight (FW). Dry weight (DW). Gallic acid equivalents (GAE). Different lowercase letters (a–c) in the same line in each parameter indicate significant differences (*p* < 0.05). The bioaccessible fractions contain between 14.1 and 15.6 mg of vegetable matrix/mL.

**Table 2:** Antiproliferative effect of microgreen bioaccessible fractions (diluted 1:10 ***v/v*** in D-MEM) after 24 h treatment on normal (CCD18-Co) and/or tumoral (Caco-2) cells.

		% Cell Viability	
		MTT Assay	
		CCD18-Co	Caco-2
Digestion Blank		102.7 ± 1.7 ^a,x^	100.9 ± 6.3 ^a,x^
Broccoli		100.8 ± 5.5 ^a,x^	90.9 ± 3.8 ^b,y^
Kale		96.9 ± 3.1 ^a,x^	89.2 ± 0.5 ^b,y^
Mustard		86.2 ± 1.2 ^b,x^	87.5 ± 2.7 ^b,x^
Radish		94.4 ± 1.3 ^ab,x^	87.8 ± 2.0 ^b,y^

Results are expressed as mean ± standard deviation (n = 4) in two independent assays. Cell viability is calculated as % with respect to digestion blank. Different lowercase letters (a,b) in the same column indicate significant differences (*p* < 0.05) among samples. Different lowercase letters (x,y) in the same line in each treatment (FBs) indicate significant differences (*p* < 0.05) between cell lines. Comparison of cell viability between control (untreated cells) vs. positive control (5-FU) for CCD18-Co (control: 101.1 ± 5.8 vs. 5-FU: 87.3 ± 5.8) and Caco-2 (control: 99.4 ± 1.96 vs. 5-FU: 76.5 ± 3.9) cells.
